# CHANGES IN SEDENTARY PATTERNS AND SELF-REPORTED HEALTH IN A RANDOMIZED CONTROLLED TRIAL OF SITTING REDUCTION

**DOI:** 10.1093/geroni/igae098.0260

**Published:** 2024-12-31

**Authors:** Dori Rosenberg, Stefani Florez Acevedo, Mikael Anne Greenwood-Hickman, Weiwei Zhu, David Arterburn, Jennifer McClure, Bev Green

**Affiliations:** Kaiser Permanente Washington Health Research Institute, Seattle, Washington, United States; University of Washington, Seattle, Washington, United States; Kaiser Permanente Washington Health Research Institute, Seattle, Washington, United States; Kaiser Permanente Washington Health Research Institute, Seattle, Washington, United States; Kaiser Permanente Washington Health Research Institute, Seattle, Washington, United States; Kaiser Permanente Washington Health Research Institute, Seattle, Washington, United States; Kaiser Permanente Washington Health Research Institute, Seattle, Washington, United States

## Abstract

**Background:**

Interventions to reduce sedentary time in older adults have been limited to feasibility studies. Building off a series of pilot studies, we aimed to test the effects of the I-STAND sitting reduction intervention to reduce sedentary patterns and improve self-reported health outcomes.

**Methods:**

283 participants were randomized to the I-STAND sitting reduction arm or an attention control condition. I-STAND included 10 brief health coach sessions, a tabletop standing desk, and fitness tracker to prompt sitting breaks. The attention control received 10 brief general healthy living focused sessions. The activPAL accelerometer worn for 7-days at baseline, 3, and 6 months measured sitting metrics. We measured pain interference, depression, loneliness and sleep disturbance by self-report. Linear regression models included baseline outcomes, covariates (e.g. demographics, health status), and whether randomization occurred pre- or post-pandemic.

**Results:**

I-STAND (N = 140) and control (N = 143) participants mean age was 68.8 (SD 6.2) years, mean BMI was 34.9 (SD 4.7) kg/m2, 51.9% had hypertension. Participants were 65.7% women, 68.9% White, 14.5% Black, 5.7% Hispanic/Latinx, 4.2% Multiracial, and 3.2% Asian. Sitting time (-31.85 mins/day, p = 0.003), prolonged sitting bouts (-.57 bouts, p = 0.004), and mean sitting bout duration (-1.8 mins, p = 0.006) reduced while standing time increased (+27.7 minutes, p = 0.003) favoring the intervention. Loneliness reduced favoring I-STAND (-0.27, 95%CI: -0.52, -0.02, p=0.032).

**Conclusion:**

I-STAND changed sitting patterns and improved loneliness. Uptake of sitting reduction interventions by older adults could improve physical and mental health.

